# Supplementary material for: A Critical View of the Application of the APEX Software (Aqueous Photochemistry of Environmentally-Occurring Xenobiotics) to Predict Photoreaction Kinetics in Surface Freshwaters
Source: Molecules. 2019 Dec 18;25(1):9. doi: 10.3390/molecules25010009 (PMC7017383; doi:10.3390/molecules25010009)
Supplement: Supplementary file 1 [file molecules-25-00009-s001.zip › 3.2.4_gcc-4.4.0.zip!/3.2.4_gcc-4.4.0/3.2.4_gcc-4.4.0/doc/octave/html/interpreter/Mathematical-Constants.html]

Mathematical Constants - Untitled


Previous: Coordinate Transformations,
Up: Arithmetic


---

### 17.8 Mathematical Constants

— Built-in Function: **e**  
— Built-in Function: **e** (n)  
— Built-in Function: **e** (n, m)  
— Built-in Function: **e** (n, m, k, ...)  
— Built-in Function: **e** (..., class)  
> Return a scalar, matrix, or N-dimensional array whose elements are all equal
> to the base of natural logarithms. The constant
> e satisfies the equation `log` (e) = 1.
>
> When called with no arguments, return a scalar with the value e. When
> called with a single argument, return a square matrix with the dimension
> specified. When called with more than one scalar argument the first two
> arguments are taken as the number of rows and columns and any further
> arguments specify additional matrix dimensions.
> The optional argument class specifies the return type and may be
> either "double" or "single".

— Built-in Function: **pi**  
— Built-in Function: **pi** (n)  
— Built-in Function: **pi** (n, m)  
— Built-in Function: **pi** (n, m, k, ...)  
— Built-in Function: **pi** (..., class)  
> Return a scalar, matrix, or N-dimensional array whose elements are all equal
> to the ratio of the circumference of a circle to its
> diameter.
> Internally, `pi` is computed as 4.0 \* atan (1.0).
>
> When called with no arguments, return a scalar with the value of
> pi.
> When called with a single argument, return a square matrix with the dimension
> specified. When called with more than one scalar argument the first two
> arguments are taken as the number of rows and columns and any further
> arguments specify additional matrix dimensions.
> The optional argument class specifies the return type and may be
> either "double" or "single".

— Built-in Function: **I**  
— Built-in Function: **I** (n)  
— Built-in Function: **I** (n, m)  
— Built-in Function: **I** (n, m, k, ...)  
— Built-in Function: **I** (..., class)  
> Return a scalar, matrix, or N-dimensional array whose elements are all equal
> to the pure imaginary unit, defined as
> `sqrt (-1)`.
> I, and its equivalents i, J, and j, are functions so any of the names may
> be reused for other purposes (such as i for a counter variable).
>
> When called with no arguments, return a scalar with the value i. When
> called with a single argument, return a square matrix with the dimension
> specified. When called with more than one scalar argument the first two
> arguments are taken as the number of rows and columns and any further
> arguments specify additional matrix dimensions.
> The optional argument class specifies the return type and may be
> either "double" or "single".

— Built-in Function: **Inf**  
— Built-in Function: **Inf** (n)  
— Built-in Function: **Inf** (n, m)  
— Built-in Function: **Inf** (n, m, k, ...)  
— Built-in Function: **Inf** (..., class)  
> Return a scalar, matrix or N-dimensional array whose elements are all equal
> to the IEEE representation for positive infinity.
>
> Infinity is produced when results are too large to be represented using the
> the IEEE floating point format for numbers. Two common examples which
> produce infinity are division by zero and overflow.
>
> ```
>           [1/0 e^800]
>           =>
>           Inf   Inf
> ```
>
> When called with no arguments, return a scalar with the value Inf.
> When called with a single argument, return a square matrix with the dimension
> specified. When called with more than one scalar argument the first two
> arguments are taken as the number of rows and columns and any further
> arguments specify additional matrix dimensions.
> The optional argument class specifies the return type and may be
> either "double" or "single".
>
>
>
> **See also:** isinf.

— Built-in Function: **NaN**  
— Built-in Function: **NaN** (n)  
— Built-in Function: **NaN** (n, m)  
— Built-in Function: **NaN** (n, m, k, ...)  
— Built-in Function: **NaN** (..., class)  
> Return a scalar, matrix, or N-dimensional array whose elements are all equal
> to the IEEE symbol NaN (Not a Number).
> NaN is the result of operations which do not produce a well defined numerical
> result. Common operations which produce a NaN are arithmetic with infinity
> (Inf - Inf), zero divided by zero (0/0),
> and any operation involving another NaN value (5 + NaN).
>
> Note that NaN always compares not equal to NaN (NaN != NaN). This behavior
> is specified by the IEEE standard for floating point arithmetic. To
> find NaN values, use the `isnan` function.
>
> When called with no arguments, return a scalar with the value NaN.
> When called with a single argument, return a square matrix with the dimension
> specified. When called with more than one scalar argument the first two
> arguments are taken as the number of rows and columns and any further
> arguments specify additional matrix dimensions.
> The optional argument class specifies the return type and may be
> either "double" or "single".
>
>
>
> **See also:** isnan.

— Built-in Function: **eps**  
— Built-in Function: **eps** (x)  
— Built-in Function: **eps** (n, m)  
— Built-in Function: **eps** (n, m, k, ...)  
— Built-in Function: **eps** (..., class)  
> Return a scalar, matrix or N-dimensional array whose elements are all eps,
> the machine precision. More precisely, `eps` is the relative spacing
> between any two adjacent numbers in the machine's floating point system.
> This number is obviously system dependent. On machines that support IEEE
> floating point arithmetic, `eps` is approximately
> 2.2204e-16 for double precision and 1.1921e-07
> for single precision.
>
> When called with no arguments, return a scalar with the value
> `eps(1.0)`.
> Given a single argument x, return the distance between x and
> the next largest value.
> When called with more than one argument the first two arguments are taken as
> the number of rows and columns and any further
> arguments specify additional matrix dimensions.
> The optional argument class specifies the return type and may be
> either "double" or "single".

— Built-in Function: **realmax**  
— Built-in Function: **realmax** (n)  
— Built-in Function: **realmax** (n, m)  
— Built-in Function: **realmax** (n, m, k, ...)  
— Built-in Function: **realmax** (..., class)  
> Return a scalar, matrix or N-dimensional array whose elements are all equal
> to the largest floating point number that is representable. The actual
> value is system dependent. On machines that support IEEE
> floating point arithmetic, `realmax` is approximately
> 1.7977e+308 for double precision and 3.4028e+38
> for single precision.
>
> When called with no arguments, return a scalar with the value
> `realmax("double")`.
> When called with a single argument, return a square matrix with the dimension
> specified. When called with more than one scalar argument the first two
> arguments are taken as the number of rows and columns and any further
> arguments specify additional matrix dimensions.
> The optional argument class specifies the return type and may be
> either "double" or "single".
>
>
>
> **See also:** realmin, intmax, bitmax.

— Built-in Function: **realmin**  
— Built-in Function: **realmin** (n)  
— Built-in Function: **realmin** (n, m)  
— Built-in Function: **realmin** (n, m, k, ...)  
— Built-in Function: **realmin** (..., class)  
> Return a scalar, matrix or N-dimensional array whose elements are all equal
> to the smallest normalized floating point number that is representable.
> The actual value is system dependent. On machines that support
> IEEE floating point arithmetic, `realmin` is approximately
> 2.2251e-308 for double precision and 1.1755e-38
> for single precision.
>
> When called with no arguments, return a scalar with the value
> `realmin("double")`.
> When called with a single argument, return a square matrix with the dimension
> specified. When called with more than one scalar argument the first two
> arguments are taken as the number of rows and columns and any further
> arguments specify additional matrix dimensions.
> The optional argument class specifies the return type and may be
> either "double" or "single".
>
>
>
> **See also:** realmax, intmin.
